# Genome-Wide Identification of Candidate Genes Associated with Antler Weight in Tahe Red Deer

**DOI:** 10.3390/ani16101424

**Published:** 2026-05-07

**Authors:** Zhengchuang Hou, Yujiao Qi, Chenchen Yang, Junjie Shao, Faling Hou, Wenxi Qian, Qinghua Gao, Chunmei Han

**Affiliations:** 1 College of Animal Science and Technology, Tarim University, Alar 843300, China; houzc0806@163.com (Z.H.); qiyujiao1128@163.com (Y.Q.); chen2yang0218@163.com (C.Y.); junjieshao191@163.com (J.S.); houfaling521@163.com (F.H.); qianwenxizj@163.com (W.Q.); 2 Key Laboratory of Animal Husbandry Science and Technology, Xinjiang Production and Construction Corps, Alar 843300, China; 3 Key Laboratory of Livestock and Forage Resources Utilization Around Tarim, Ministry of Agriculture and Rural Areas, Alar 843300, China

**Keywords:** Tahe red deer, genetic diversity, antler weight, genome-wide association study, candidate gene

## Abstract

The Tahe red deer is derived from the wild Tarim red deer, an endemic subspecies native to the Tarim Basin in Xinjiang, China. It has recently received official approval as a locally bred deer variety, developed through artificial breeding programs. This breed retains several advantageous traits from its wild ancestors, including tolerance to coarse forage, drought resistance, and a high yield of velvet antlers. To investigate the genetic mechanisms underlying velvet antler production, phenotypic data for antler weight and blood samples were collected from 73 adult Tahe red deer. Whole-genome sequencing and genome-wide association analysis were performed to identify genetic variants associated with antler weight. Population genetic analysis revealed that the observed heterozygosity (Ho) and expected heterozygosity (He) were 0.31291 and 0.32832, respectively, while the nucleotide diversity (π) was 2.17 × 10^−3^, indicating relatively high genetic diversity within the Tahe red deer population. Using a mixed linear model (MLM), a total of 189 candidate genes and 1387 significant SNP loci associated with antler weight were identified (*p* < 1.0 × 10^−5^). Gene Ontology (GO) enrichment analysis revealed that these candidate genes are primarily involved in intracellular calcium ion homeostasis, peptide and protein biosynthesis, extracellular matrix organization, the regulation of glycolysis, and cytoskeleton-related processes, including actin filaments and microfibrils. These biological functions are closely related to cell proliferation, differentiation, energy metabolism, and tissue remodeling. The Kyoto Encyclopedia of Genes and Genomes (KEGG) pathway analysis further indicated that the candidate genes are significantly enriched in several pathways, including the Notch signaling pathway, the cGMP–PKG signaling pathway, the regulation of the actin cytoskeleton, ribosome biogenesis, and mucin type O-glycan biosynthesis. These results suggest that these genes may participate in velvet antler growth and development by regulating cell proliferation and differentiation, cytoskeletal remodeling, and protein synthesis. Overall, this study identifies SNP loci and candidate genes significantly associated with antler weight in Tahe red deer, providing a theoretical basis for genetic improvement and marker-assisted selection for velvet antler production in this breed.

## Simple Summary

The Tahe red deer of Xinjiang is an elite deer breed developed over more than 40 years of artificial domestication and systematic selection, based on the wild Tarim red deer population. Characterized by large stature, rapid growth, and high antler yield, the herd size is approximately 40,000 individuals. Each deer produces an average of 7 kg of antlers per harvest, with annual yields exceeding 10 kg. This breed serves as a vital pillar of the distinctive livestock industry in southern Xinjiang. Domestically and internationally, red deer antlers are widely utilized in pharmaceuticals, health supplements, cosmetics, and other sectors as a high-value-added product. Current genetic research on antler weight in Tahe red deer primarily focuses on transcriptional characteristics during antler development, with no comprehensive genome-wide analysis of the genetic basis for antler weight. This study employs whole-genome sequencing to identify candidate genes and their associated SNPs that are significantly correlated with antler weight. It aims to elucidate the genetic characteristics and mechanisms underlying red deer antler development while providing theoretical support for molecular marker-assisted selection and precision breeding in Tahe red deer.

## 1. Introduction

The Tarim red deer (*Cervus elaphus yarkandensis*) is a large subspecies of deer endemic to China, primarily distributed along the Tarim River and its tributaries within the Tarim Basin of the Xinjiang Uygur Autonomous Region. The Tahe red deer is derived from wild Tarim red deer and has undergone systematic domestication and artificial selection since 1958, officially becoming Xinjiang’s first local breed in 2024. This subspecies is characterized by its high antler yield, tolerance for coarse feed, drought resistance, and strong resilience. The current population of Tahe red deer is approximately 40,000 individuals, and they are primarily farmed in the Korla region of Xinjiang. This breed plays a vital role in the local livestock production system and contributes to distinctive economic development [1].

Over the past two decades, research has intensely focused on the rapid growth and regeneration of deer antlers. It is now confirmed that this process relies on the proliferation and differentiation of antler stem cells. Specifically, antler growth is sustained through two mechanisms: endochondral ossification and intramembranous ossification, both facilitated by these stem cells. Substantial findings have emerged from molecular regulatory mechanisms. For instance, transcriptomic analysis of antler tissue from sika deer during the growth phase, conducted from 2022 to 2025, identified approximately 14,000 differentially expressed genes. These genes are primarily distributed across various antler growth stages and tissues, participating in biological processes such as cell proliferation, stem cell self-renewal, cartilage formation, osteogenic differentiation, angiogenesis, and mineralization. Among these, transcription factors such as *MYC*, *KLF4*, *NFE2L2*, and *JDP2* primarily regulate cell proliferation and regenerative capacity, while *PRDM1*, *FOSL1*, *BACH1*, and *NFATC1* are closely associated with cell differentiation, bone remodeling, and tissue regeneration regulation. This reveals the complex molecular regulatory network underlying the rapid growth and regeneration of deer antler velvet [2,3,4]. In 2021, Jia et al. analyzed the transcriptome and mRNA of Gansu red deer antlers, identifying genes including *COL2A1*, *SOX9*, *WWP2*, *FGFR1*, *SPARC*, and *LOX* as participants in the growth of red deer antlers during the osteogenic stage [5]. In 2025, Chen et al. [6] utilized the transcriptome of regenerated Tarim red deer antler tissue to identify 56 differentially expressed genes that influence stem cell proliferation through signaling pathways such as Hedgehog and Wnt. Among these genes, the *BVES* gene plays a critical role in maintaining the steady-state balance between proliferation and differentiation in antler mesenchymal stem cells by regulating the Wnt/β-catenin signaling pathway [6]. In 2025, Ba et al. [7] identified RXFP2-positive mesenchymal stem cells in the antlerogenic periosteum. These cells demonstrate strong proliferative and differentiation capacities, as well as the ability to differentiate into cartilage and bone tissues. They play a direct role in the postnatal growth and development of antlers, thereby clarifying the key stem cell source underlying antler regeneration [7]. This regulation is essential for sustaining the rapid and normal growth and development of red deer antlers. However, transcriptome data only reflect gene functional characteristics at specific developmental stages or physiological states, thereby limiting a comprehensive analysis of genome-wide genetic variation associated with antler traits. Therefore, conducting a genome-wide analysis based on whole-genome sequencing data is crucial for revealing the genetic architecture of antler growth and identifying key functional genes.

Genome-wide association studies (GWAS) are widely regarded as a powerful approach for uncovering the genetic architecture of economically important traits. By enabling systematic identification of trait-associated loci across the genome, GWAS has become an essential tool for advancing selective breeding in livestock and poultry. In 2023, Teng et al. conducted a GWAS analysis on longitudinal phenotypes of Chinese Holstein dairy cows, identifying multiple significantly associated genetic regions and candidate genes (e.g., *DGAT1*, *ABCG2*, *GHR*), thereby providing crucial molecular marker resources for genomic selection and molecular breeding of milk production and composition traits [8]. In 2025, Ni et al. performed a GWAS using whole-genome sequencing data from 1004 chickens, identifying 5982 SNPs associated with egg production traits and three core candidate genes [9]. Regarding deer genome research, in 2017, Hu et al. collected antler weight data from 100 sika deer individuals and analyzed whole-genome sequencing data, identifying the *OAS2* and *ALYREF/THOC4* genes as significantly associated with antler weight traits [10]. In 2022, the research group integrated genomic and transcriptomic data, combining GWAS with weighted gene co-expression network analysis. They identified key gene modules and pathways significantly associated with rapid antler growth in sika deer. The *IGF1R* module was found to primarily regulate the proliferation and differentiation of antler trophoblast cells, while the *LOX* module participated in collagen formation and growth stability across antler tissues [11]. Collectively, these studies preliminarily indicate that antler-related traits exhibit typical polygenic regulatory genetic characteristics, with candidate genes predominantly enriched in pathways related to bone formation, cell proliferation, and metabolism. However, genome-wide association studies focusing on antler weight traits in Tarim red deer have yet to be conducted, and their underlying genetic regulatory mechanisms remain to be systematically elucidated.

This research performed a Genome-Wide Association Study (GWAS) using whole-genome resequencing data combined with phenotypic data regarding antler weight from 73 artificially bred Tahe red deer. Through the integration of genetic diversity assessments and population structure analysis with functional annotation and pathway enrichment evaluations, we systematically pinpointed significant genetic loci and potential candidate genes that impact antler growth. The findings offer critical data support for future genetic improvement and selective breeding of antler traits in Tarim red deer.

## 2. Materials and Methods

### 2.1. Blood Sampling and Phenotypic Data Collection

At the Second Division of the Xinjiang Production and Construction Corps’ Deer Farm, a total of 73 adult male Tahe red deer, ranging in age from 3 to 12 years, were selected for the purpose of phenotyping antler weight. Fresh antlers with three tines were weighed immediately after sawing, according to standard antler classification criteria. At the same time, 5 mL of venous blood was collected from each individual for subsequent genomic analysis.

### 2.2. DNA Extraction and Whole-Genome Resequencing

Genomic DNA was isolated from blood specimens utilizing the Tiangen Blood Genomic DNA Extraction Kit (DP304,TIANGEN Biotech Co., Ltd. Beijing, China), adhering to the instructions provided by the manufacturer. DNA samples that fulfilled the quality criteria were then employed for library construction and sequencing. Whole-genome sequencing took place on the BGI DNBSEQ platform (Shenzhen, China), achieving an average sequencing depth of around 10×.

### 2.3. Genotyping and Quality Control

Raw sequencing reads were filtered for quality using fastp v0.23.2 [12]. Clean reads were then aligned to the Tarim red deer reference genome (GCA_010411085.1, https://www.ncbi.nlm.nih.gov/datasets/genome/GCA_010411085.1/, accessed on 4 May 2026) using BWA-MEM2 v2.2.1 [13,14]. The resulting alignment files were converted to BAM format and sorted using SAMtools v1.9 with default parameters [15]. Duplicate reads were detected and eliminated through the use of the MarkDuplicates module available in GATK v4.1.4. Following this, variant calling was executed utilizing the Genome Analysis Toolkit (GATK v4.1.4). For variant filtering, the VariantFiltration module was applied with the subsequent parameters: QD < 2.0 || FS > 60.0 || MQ < 40.0 || SOR > 3.0 || MQRankSum < −12.5 || ReadPosRankSum < −8.0 [16]. Biallelic loci located on autosomes were extracted using BCFtools v1.20 [14]. Further quality control was performed using PLINK v1.9 with the parameters --geno 0.1, --hwe 1 × 10^−7^, --maf 0.05, and --mind 0.15, retaining SNPs with a minor allele frequency (MAF) ≥ 0.05 [17].

### 2.4. Group Structure Analysis

Principal component analysis (PCA) was performed using PLINK v1.9 with the command plink --vcf A.vcf --pca. The identity-by-state (IBS) similarity matrix among individuals was calculated using SNPRelate v1.40 to assess the population kinship structure [18]. Pairwise genetic distances (p-distance) were calculated using VCF2Dis v1.53 with the command VCF2Dis -vcf A.vcf -out pDist.mat [19]. Based on the resulting distance matrix, a neighbor-joining (NJ) phylogenetic tree was constructed, and the tree was visualized and annotated using iTOL v7.5.

### 2.5. Genetic Diversity and Linkage Disequilibrium Analysis

PLINK v1.90 was used to calculate genetic diversity parameters, including observed heterozygosity (Ho), expected heterozygosity (He), and nucleotide diversity (π). Linkage disequilibrium (LD) was estimated as the squared correlation coefficient (r^2^) using PopLDdecay v3.40. SNP pruning was conducted with the parameter -indep-pairwise 25 5 0.2 [20].

### 2.6. Genome-Wide Association Analysis and Visualization

Association analysis between antler weight and SNP markers in the Tahe red deer population was conducted using a mixed linear model (MLM) implemented in GEMMA v0.98.4 [21]. Age was included in the model as a fixed-effect covariate. To account for population stratification and genetic relatedness among individuals, the first three principal components (PC1–PC3) derived from principal component analysis (PCA) were incorporated as fixed effects. Additionally, a kinship matrix constructed from genome-wide SNP data was fitted as a random effect to address background genetic similarity. The genome-wide significance threshold was established at −log10(*p*-value) = 5. The mixed linear model can be expressed as follows:

y = Xβ + Zu + ε,u ~ N(0, K), ε ~ N(0, I)(1)

In the model, y represents the vector of antler weight phenotypes; X denotes the design matrix for fixed effects, including SNP effects, PC1–PC3, and age as a covariate; and β corresponds to the vector of fixed-effect coefficients. Z is the design matrix for random effects, u represents the random genetic effects modeled using the kinship matrix (K), and ε denotes the residual error.

To further control for false positives arising from multiple testing, the *p*-values of all SNPs were adjusted using both Bonferroni correction and false discovery rate (FDR) correction, which were implemented via the p.adjust function in R. Additionally, Manhattan plots and Q–Q plots were generated using the CMplot package in R v4.4.3 [22].

### 2.7. Functional Enrichment Analysis of Candidate Genes and Construction of Protein Interaction Networks

Functional annotation of protein sequences from the Tahe red deer reference genome (GCA_010411085.1; file: GCA_010411085.1_protein.fna) was performed using the online DAVID v2025_2 database, generating annotation files containing corresponding Gene Ontology (GO) and Kyoto Encyclopedia of Genes and Genomes (KEGG) identifiers [23]. GO and KEGG enrichment analyses were subsequently conducted using the DAVID functional annotation tool. Candidate genes located at significant SNP loci were defined as the target gene set, while the annotated gene set served as the background dataset.

The STRING database was utilized to conduct an analysis of protein–protein interactions (PPI) in order to explore possible functional interactions between the candidate genes. The resulting interaction network was visualized using Cytoscape software v3.10.2.

The STRING database was utilized to conduct an analysis of protein–protein interactions (PPIs) for examining the functional relationships between genes, and Cytoscape software was employed to visualize the resulting network.

### 2.8. Validation ofDEGs by qRT-PCR

To ensure the dependability of the GWAS findings, four candidate genes—*MEF2D*, *LTBP1*, *ADAMTS20*, and *ITGB6*—were chosen at random for quantitative real-time reverse transcription PCR (RT-qPCR), using *GAPDH* (NM_001034034.2) as the reference gene. Six 5-year-old adult male Tahe red deer were selected, including three individuals from the high-yield group (annual antler weight ≥ 7 kg) and three from the low-yield group (annual antler weight < 4 kg). Antler tissue samples were collected at 70–76 days after casting, corresponding to the rapid growth phase. At this stage, chondrocyte proliferation, angiogenesis, and endochondral ossification are highly active, representing a key developmental window that largely determines the final size and mass of the antler.

Total RNA was extracted from the velvet, mesenchymal stem cells, cartilage, and bone tissues using TRIzol reagent (ER501-01-V2, Beijing TransGen Biotech Co., Ltd., Beijing, China). Gene-specific primers for *MEF2D*, *LTBP1*, *ADAMTS20*, *ITGB6*, and *GAPDH* were designed using Primer Premier 6 software, and the primer sequences used for RT-qPCR are listed in Table 1. First-strand cDNA was synthesized using the HyperScript III RT SuperMix for qPCR with gDNA Remover kit (R202-02, Shanghai EnzyArtisan Biotech Co., Ltd., Shanghai, China).

The amplification of RT-qPCR was conducted following the manufacturer’s guidelines using the 2× S6 Universal SYBR qPCR Mix Kit (Q204, Shanghai EnzyArtisan Biotech Co., Ltd., Shanghai, China) on a Q2000 Real-Time PCR System (Hangzhou LongGene Scientific Instruments Co., Ltd., Hangzhou, China). The thermal cycling conditions were as follows: initial denaturation at 95 °C for 30 s, followed by 41 cycles of denaturation at 95 °C for 10 s and annealing/extension at 60 °C for 30 s. Relative gene expression levels were calculated using the 2^−ΔΔCt^ method. All RT-qPCR assays were performed with three biological replicates, each including three technical replicates. Differences in gene expression among groups were analyzed using one-way analysis of variance (ANOVA), followed by Tukey’s multiple comparison test as a post hoc analysis when significant effects were detected. A value of *p* < 0.05 was considered statistically significant. All statistical analyses were conducted using GraphPad Prism v10.1.2.

## 3. Results

### 3.1. SNP Identification and Filtering Results

A total of 21,198,712 raw SNPs were detected using the GATK HaplotypeCaller v4.1.4. Following rigorous filtering, which included the removal of sites with high missing rates, insufficient sequencing depth, violations of Hardy–Weinberg equilibrium (HWE), and non-bivalent loci, 11,467,176 high-quality SNPs were ultimately obtained for subsequent analysis.

### 3.2. Genetic Diversity and Population Structure Analysis

An examination of genetic diversity metrics showed that the observed heterozygosity (Ho) for Tahe red deer was 0.31291, while the expected heterozygosity (He) stood at 0.32832, and the nucleotide diversity (π) was 2.17 × 10^−3^. These findings suggest that the genetic diversity within the Tahe red deer population is comparatively high.

**Figure 1:**
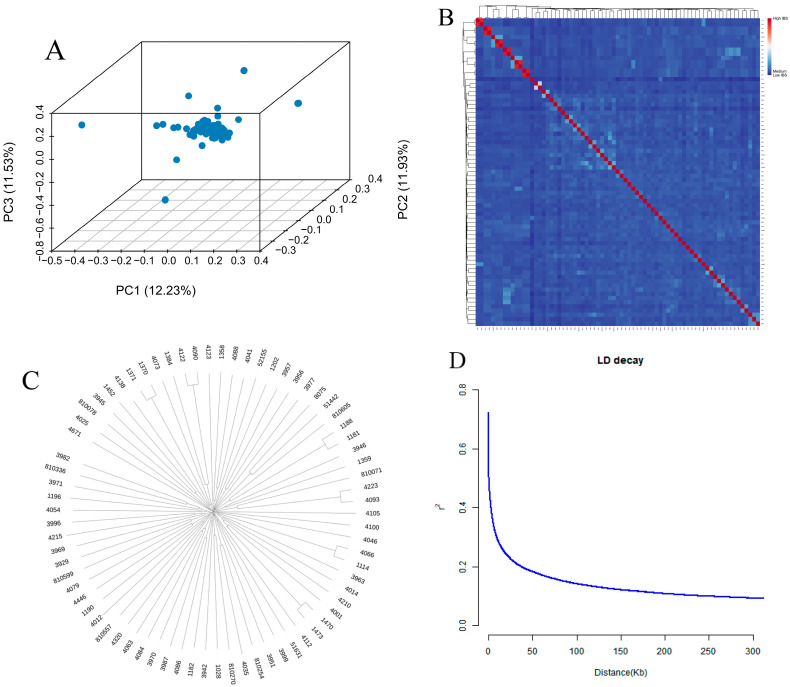
Population structure of the Tahe red deer. (**A**) Principal component analysis (PCA). (**B**) Heatmap of the kinship matrix. (**C**) Neighbor-joining tree of the Tarim red deer. (**D**) Linkage disequilibrium (LD) decay plot.

The genetic structure and phylogenetic relationships of 73 Tahe red deer were analyzed using Principal Component Analysis (PCA), a neighbor-joining tree (NJ tree), and ADMIXTURE. Results from the PCA of whole-genome SNP data revealed a dispersed overall sample structure with a trend toward population differentiation (Figure 1A). The heatmap indicated weak phylogenetic relationships among individuals within the population, with no distinct hierarchical clustering observed, suggesting a uniform sample origin (Figure 1B). The phylogenetic tree indicated that the 73 Tahe red deer formed three major groups, each comprising multiple individuals (Figure 1C). Linkage disequilibrium (LD) decay analysis revealed a significant decline in the pairwise LD (r^2^) between single nucleotide polymorphisms (SNPs) as the physical distance increased. Within the initial 50 kb, r^2^ exhibited a rapid decrease, dropping to approximately 0.2 at around 50 kb. Beyond this threshold, the rate of decline slowed considerably and stabilized at a low level (Figure 1D). This result indicates that SNPs within an approximately 50 kb region still maintain a certain degree of linkage, suggesting that 50 kb represents the LD decay distance in the Tahe red deer population.

### 3.3. Genome-Wide Association Analysis

**Figure 2:**
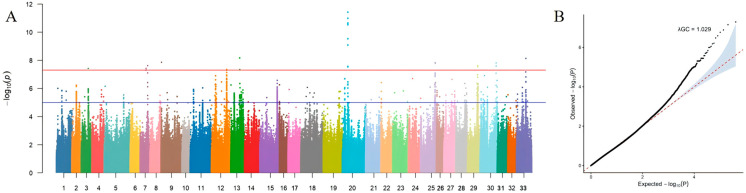
Manhattan and quantile-quantile plot of SNP GWAS. (**A**) Manhattan plot of the GWAS results. (**B**) Quantile–quantile (Q–Q) plot of the GWAS results.

The Manhattan plot and Q–Q plot showed consistent results. After Bonferroni and FDR correction, a total of 1387 significant SNPs reached the threshold of −log_10_(P) = 5 (Supplementary Table S1). The Q–Q plot indicated that the −log10(P) values of most SNPs were highly consistent with the expected distribution, with data points closely aligned along the reference diagonal line and only slight deviation observed in the tail region (Figure 2A,B).

Candidate genes associated with the antler weight trait were identified by analyzing the significant SNP sites and their upstream and downstream 50 kb regions, resulting in the annotation of a total of 189 candidate genes.

### 3.4. GO and KEGG Pathway Enrichment Analyses

**Figure 3:**
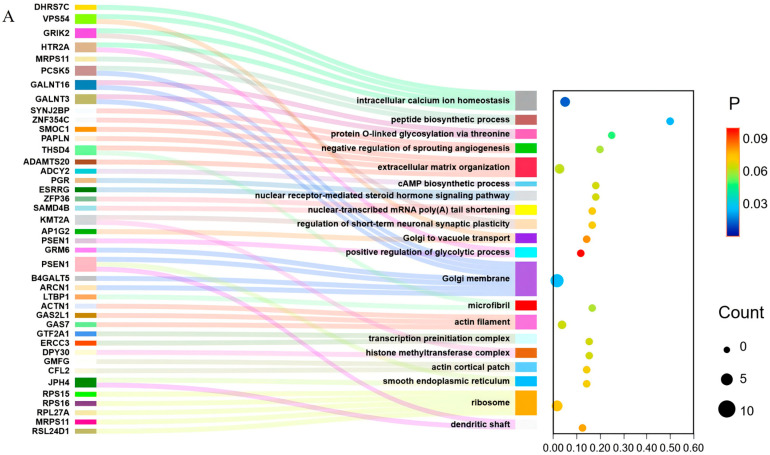

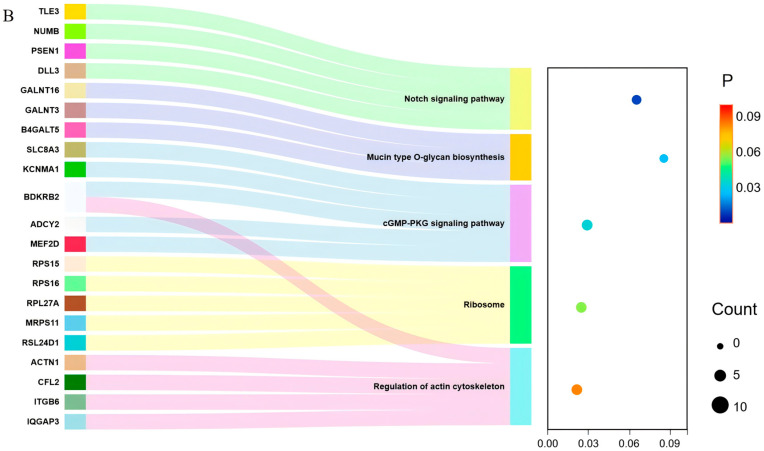
Functional enrichment analysis of candidate genes. (**A**) Gene Ontology (GO) enrichment analysis of candidate genes. (**B**) Kyoto Encyclopedia of Genes and Genomes (KEGG) pathway enrichment analysis of candidate genes.

GO and KEGG enrichment analyses revealed that 189 candidate genes were significantly enriched (*p* < 0.05) in 20 Gene Ontology (GO) terms and 5 Kyoto Encyclopedia of Genes and Genomes (KEGG) pathways (Figure 3A,B). The findings from the GO enrichment analysis suggest that the candidate genes are mainly concentrated in categories including extracellular matrix organization, signaling pathways mediated by nuclear receptors for steroid hormones, maintenance of intracellular calcium ion homeostasis, inhibition of sprouting angiogenesis, and the formation of microfibrils. Moreover, the results of KEGG enrichment indicate that the candidate genes show significant enrichment in various pathways, such as the Notch signaling pathway, biosynthesis of Mucin type O-glycans, cGMP-PKG signaling pathway, ribosome biogenesis, and the regulation of the actin cytoskeleton.

**Figure 4:**
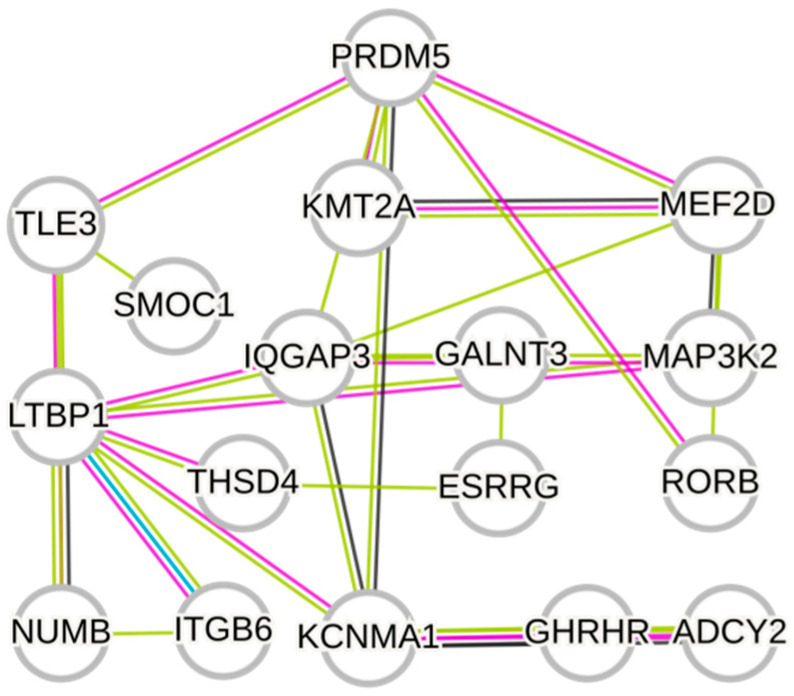
Protein–protein interaction (PPI) network of candidate genes.

By integrating the biological functions of associated genes with significance analysis, we analyzed information on 20 genes, including *ADCY2*, *NUP153*, *KIF13A*, *ADAMTS20*, and *SNX6* (see Table 2). A protein interaction network was constructed, revealing extensive functional associations among the associated genes, which together form a well-structured regulatory network framework. These genes serve as pivotal hubs within the regulatory network through their interactions with multiple signaling pathways, including WNT, NOTCH, MAPK, and PI3K-AKT (see Figure 4).

### 3.5. Candidate Gene Validation

**Figure 5:**
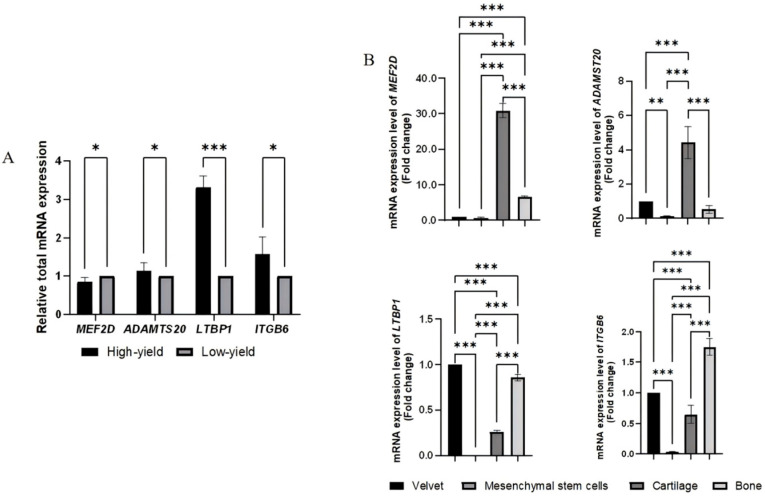
Expression Analysis of *ADAMTS20*, *LTBP1*, *ITGB6*, and *MEF2D* Across Antler Tissue Layers. (**A**) Relative expression levels of *ADAMTS20*, *LTBP1*, *ITGB6*, and *MEF2D*. (**B**) Expression profiles of four genes across antler tissue layers (velvet, mesenchymal stem cells, cartilage, and bone). Statistical significance levels are denoted by asterisks as follows: *** *p* < 0.001; ** *p* < 0.01; * *p* < 0.05; and no label indicates *p* ≥ 0.05.

Randomly selected genes *ADAMTS20*, *LTBP1*, *ITGB6*, and *MEF2D* were evaluated. Real-time quantitative PCR analysis indicated that these genes were expressed in the velvet, mesenchymal stem cells, cartilage, and bone tissues of Tahe red deer antlers at 70–76 days post-growth. Notably, expression levels were significantly higher in high-yield group antlers compared to low-yield group antlers (*p* < 0.01) (Figure 5A). Among these genes, *LTBP1* and *ITGB6* demonstrated extremely significantly higher expression levels in velvet and bone tissues relative to other tissues (*p* < 0.01), whereas *ADAMTS20* and *MEF2D* exhibited significantly higher expression in cartilage tissue compared to other tissues (*p* < 0.05) (Figure 5B).

## 4. Discussion

**Figure 6:**
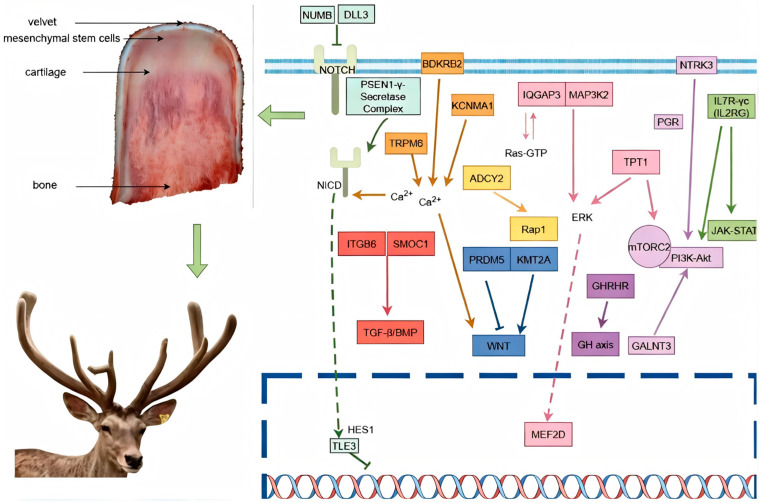
Interaction network of selected candidate genes and associated pathways (by Figdraw 2.0). Arrows (→) indicate activation or promotion, T-shaped lines (⊣) indicate inhibition or negative regulation, and the dashed box indicates processes occurring within the nucleus.

Deer antler growth is initiated by the continuous proliferation of mesenchymal stem cells at the antler tip, forming a stem cell niche. These cells subsequently undergo chondrogenic differentiation, and through endochondral ossification, enable the rapid longitudinal extension of the antler. The associated genes identified by the institute, along with their significantly correlated SNP sites, interact across multiple pathways to achieve this biological process (Figure 6).

Based on the LD decay results, the decay distance in the Tahe red deer was approximately 50 kb, indicating that the ±50 kb region around a mutation site represents the average linkage range in this population. Because the LD decay distance is one of the important criteria for determining the genetic information of loci adjacent to trait-associated sites [24], we extended 50 kb upstream and downstream from each significant SNP as the search interval during candidate gene annotation. Using this approach, a total of 189 candidate genes were identified. On this basis, genes annotated within the significant SNP regions were further prioritized using three criteria: (1) documented functional relevance to biological processes such as skeletal development, chondrogenesis, cell proliferation, and angiogenesis; (2) the statistical significance of the SNPs and the strength of the association signals in their surrounding regions; and (3) involvement in enriched GO terms and KEGG pathways. From the 189 candidate genes identified, 20 representative genes were selected for detailed discussion. Among these, *IQGAP3*, *MAP3K2*, *LTBP1*, *TPT1*, *IL7R*, *NUMB*, *TLE3*, *PRDM5*, *RORB*, and *KMT2A* are closely associated with the regulation of stem cell proliferation and differentiation. Research indicates that *IQGAP3* promotes cell cycle progression by enhancing the Ras–ERK signaling pathway, thereby sustaining stem cell proliferation [25]; *MAP3K2* maintains stem cell niche stability by regulating the expression of stem cell factors, such as R-spondin1 [26]. *LTBP1* likely regulates TGF-β signaling pathways to uphold mesenchymal stem cell proliferation and fate homeostasis [27]. Furthermore, *TPT1* plays a crucial role in the survival and proliferation of neural progenitor cells [28], *IL7R* can coordinate progenitor cell proliferation, differentiation, and gene rearrangement by activating downstream signaling pathways such as JAK/STAT5, thereby maintaining the expansion and fate determination of early lymphoid progenitor cells [29]. Regarding stem cell differentiation, *NUMB* regulates progenitor cell proliferation and differentiation by inhibiting Notch1 signaling [30], *TLE3* promotes the exit from pluripotency and initiates differentiation in embryonic stem cells by suppressing the expression of pluripotency genes [31], *PRDM5* participates in chromatin architecture regulation by interacting with insulator-binding proteins, thereby modulating the transcriptional state of embryonic stem cells and influencing their self-renewal capacity and differentiation potential [32]. Concurrently, *RORB* drives lineage differentiation in neural progenitor cells [33], *KMT2A* can regulate the expression of development-related genes during the growth and regeneration of tissues such as bone and hematopoietic systems, thereby maintaining the rapid proliferative state of skeletal mesenchymal progenitor cells and hematopoietic progenitor cells [34]. These genes primarily contribute to stem cell proliferation and differentiation by regulating key signaling pathways and transcriptional networks. As the only organ in mammals capable of complete periodic regeneration, antler growth relies on the sustained proliferation and differentiation of apical mesenchymal stem cells (MSCs) within the antler, forming a stable stem cell pool that continuously differentiates into cartilage and bone tissues. The candidate genes identified in this study, which are associated with stem cell proliferation and differentiation, may regulate the proliferative capacity and lineage differentiation of antler apical mesenchymal stem cells. This regulation likely maintains the cellular source and developmental potential necessary for rapid antler regeneration, thereby significantly influencing both antler growth and velvet yield formation.

Among the genes annotated at significant SNP loci, *MEF2D*, *SMOC1*, *THSD4*, *ITGB6*, *GALNT3*, *ESRRG*, *GHRHR*, *KCNMA1*, and *TRPM6* are closely associated with chondrogenesis and bone differentiation processes. Studies indicate that *MEF2D* is highly expressed in chondrocytes during the hypertrophic phase, promoting chondrocyte maturation and activating osteogenesis-related genes to drive endochondral ossification [35]. *SMOC1*, a downstream target gene of *Runx2*, regulates the extracellular matrix and BMP/TGF-β signaling pathways, promoting the differentiation of mesenchymal stem cells into chondrocytes and maintaining cartilage template formation [36]. *THSD4*, a TGF-β-associated extracellular matrix protein, plays a role in regulating cartilage phenotype stability and chondrogenesis [37]. *ITGB6* supports the chondrogenic differentiation of mesenchymal stem/progenitor cells and suppresses abnormal chondrocyte hypertrophy by activating latent TGF-β and enhancing TGF-β/SMAD signaling [38]. *GALNT3* regulates *FGF23* activity through O-glycosylation, thereby influencing chondrocyte metabolism and differentiation [39,40]. In the context of bone differentiation, *ESRRG* negatively regulates osteoblast differentiation and bone formation by inhibiting the BMP2–Runx2 osteogenic signaling pathway [41]; *GHRHR* (GHSR), as the receptor for ghrelin, mediates the regulatory effects of ghrelin on osteoblast function and participates in bone remodeling and bone metabolism [42]; *KCNMA1* affect osteoblast differentiation and bone mineralization by regulating Ca^2+^ signaling and ion transport, respectively [43]; *TRPM6* maintains magnesium homeostasis to support bone mineralization and osteocyte function, thereby indirectly influencing bone metabolism and the maintenance of bone mineral density, and playing an important regulatory role in skeletal health [44]. Furthermore, *ITGB6* enhances the osteogenic differentiation of mesenchymal stem cells by activating latent TGF-β and amplifying TGF-β/SMAD signaling [45]. During antler growth, mesenchymal stem cells at the antler apex initially differentiate into chondrocytes to form a cartilage template, which is subsequently replaced by bone tissue through endochondral ossification, facilitating rapid longitudinal elongation of the antler. Consequently, the candidate genes identified in this study, which are associated with chondrogenesis and bone differentiation, may promote rapid growth and structural remodeling of antler tissue by regulating cartilage formation and subsequent ossification processes, thereby significantly impacting the antler growth rate and yield.

GO and KEGG enrichment analyses showed that the candidate genes were mainly enriched in extracellular matrix organization, microfibril, actin filament, and regulation of actin cytoskeleton, suggesting that extracellular matrix remodeling and cytoskeletal regulation may participate in cell migration and tissue construction during antler growth, thereby supporting cartilage template formation and subsequent ossification. Enrichment in the Notch signaling pathway, nuclear receptor-mediated steroid hormone signaling pathway, and cAMP biosynthetic process indicates that stem cell differentiation and hormone signaling may jointly contribute to antler tissue development. The enrichment of intracellular calcium ion homeostasis, negative regulation of sprouting angiogenesis, and the cGMP-PKG signaling pathway further suggests important regulatory roles of calcium signaling and angiogenesis during the rapid growth and ossification stages of antler development. Overall, these pathways form an interactive network that promotes antler growth by coordinating stem cell regulation, cell migration, matrix construction, vascular support, and protein synthesis.

The reliability of GWAS results can be affected by several factors, including population differences, genetic relatedness among individuals, the number of genetic markers and linkage disequilibrium, as well as trait complexity and phenotypic measurement error [46]. In this study, the Tahe red deer population exhibited relatively high genetic diversity (Ho = 0.31291, He = 0.32832, π = 2.17 × 10^−3^), and the similarity between Ho and He indicates that the population is close to a random mating state. The IBS heatmap based on genome-wide SNPs showed no obvious family structure or close kinship among the 73 samples, suggesting a high level of genetic independence among individuals. Therefore, the influences of population differences and genetic relatedness were relatively low in this study population. On this basis, a mixed linear model incorporating both population structure and kinship information was applied to further reduce false-positive results. The favorable genetic characteristics of the population, together with statistical correction, effectively reduced the potential false-positive risk associated with the relatively small sample size and ensured the reliability and biological interpretability of the GWAS results.

## 5. Conclusions

This study identified 1387 significant SNP loci through GWAS analysis and annotated 189 candidate genes significantly associated with antler weight traits in the Tahe red deer. Among these, 20 key genes, including *IQGAP3*, *MAP3K2*, and *LTBP1*, along with their associated signaling pathways, were found to directly regulate biological processes such as the proliferation and differentiation of antler mesenchymal stem cells, as well as chondrogenesis and osteogenic differentiation. These findings provide valuable genomic resources and theoretical support for the genetic improvement and selective breeding of antler weight traits in Tahe red deer.

## Figures and Tables

**Table 1:** Candidate Gene Primer Information.

Primer Name	Primer Sequence	Annealing Temperature (°C)	Fragment Length (bp)
*MEF2D*	F: GCTCTGTTCTTCTCAGAGTCCTT R: GTCGGTTCCGCTCATCAGT	60	260
*LTBP1*	F: GACACGGCAGGCAAGCGATG R: GGTGGGACACAGCTTGGTTTGG	60	184
*ITGB6*	F: TCCCCACCTTCTTGGTGATTG R: CCGTTTCAGTTCTCGCTGGA	60	123
*ADAMTS20*	F: ACAATGGCGATATGGTTCT R: CTGGCTGATGATAGTTGATG	60	260
*GAPDH*	F: TGTTTGTGATGGGCGTGAACCA R: ATGGCGTGGACAGTGGTCATAA	60	154

**Table 2:** List of candidate genes identified through GWAS analysis.

Gene Name	Number of Genes	Chromosome	*p*-Value
*KMT2A*	1	1	1.468324 × 10^−6^
*ADAMTS20*	1	3	3.805432 × 10^−8^
*LTBP1*	1	11	1.668161 × 10^−6^
*SMOC1*, *NUMB*, *TLE3*, *THSD4*	4	12	≤7.263909 × 10^−6^
*ESRRG*	1	14	9.024180 × 10^−6^
*KCNMA1*	1	15	4.445326 × 10^−6^
*PRDM5*	1	17	1.182999 × 10^−6^
*GHRHR*	1	18	4.084938 × 10^−6^
*IQGAP3*, *MEF2D*	2	20	≤2.221995 × 10^−6^
*IL7R*	1	25	7.996876 × 10^−7^
*TRPM6*, *RORB*	2	29	4.348767 × 10^−7^
*TPT1*, *MAP3K2*, *GALNT3*, *ITGB6*	4	33	≤2.145583 × 10^−6^

## Data Availability

All datasets generated in this study are available from the corresponding author upon reasonable request.

## Supplementary Materials

The following supporting information can be downloaded at: https://www.mdpi.com/article/10.3390/ani16101424/s1, Table S1: List of genes annotated based on GWAS results.

## Abbreviations

The following abbreviations are used in this manuscript:

GWAS	Genome-Wide Association Studies
PCA	Principal Component Analysis
Ho	Observed Heterozygosity
He	Expected Heterozygosity
Π	Nucleotide Polymorphism
LD	Linkage Disequilibrium
MLM	Mixed Linear Model
Q-Q	Quantile-Quantile
PPI	Protein-Protein Interactions
MSCs	Mesenchymal Stem Cells
